# Complete genome sequence of a yellow-pigmented *Pantoea* sp. XAF26B01_ASV70 isolated from blue-winged warbler feces

**DOI:** 10.1128/mra.00121-26

**Published:** 2026-06-22

**Authors:** Sebastian Gallego, Alix E. Matthews, Victoria Gates, Kartikey Sharma, Marcella D. Baiz

**Affiliations:** 1 Department of Biological Sciences, University at Buffalo, State University of New York 12292 , Buffalo, New York, USA; University of Wisconsin-Madison, Madison, Wisconsin, USA

**Keywords:** *Pantoea*, genome, carotenoids, birds, gut microbiome

## Abstract

We report the genome assembly of a yellow-pigmented *Pantoea* sp. isolated from blue-winged warbler feces in Western New York, USA. Long read sequencing yielded a complete circular chromosome and three circular plasmids. The largest plasmid encodes a carotenoid biosynthesis (*crt*) gene cluster consisting of *crtE, crtX, crtY, crtI,* and *crtB*.

## ANNOUNCEMENT

*Pantoea* is a genus of yellow-pigmented, gram-negative bacteria in the family Erwiniaceae (1). *Pantoea* synthesize carotenoid pigments, which are antioxidants that protect against reactive oxygen species, UV radiation, and act as virulence factors (2). In a previous gut microbiome study, *Pantoea* was recovered as a core bacterial taxon across multiple species of warblers (3). Here, we assemble the genome of a *Pantoea* isolate to investigate the genetic basis of carotenoid biosynthesis and begin to understand functional traits that may explain its prevalence in warbler gut microbiomes.

**Fig 1:**
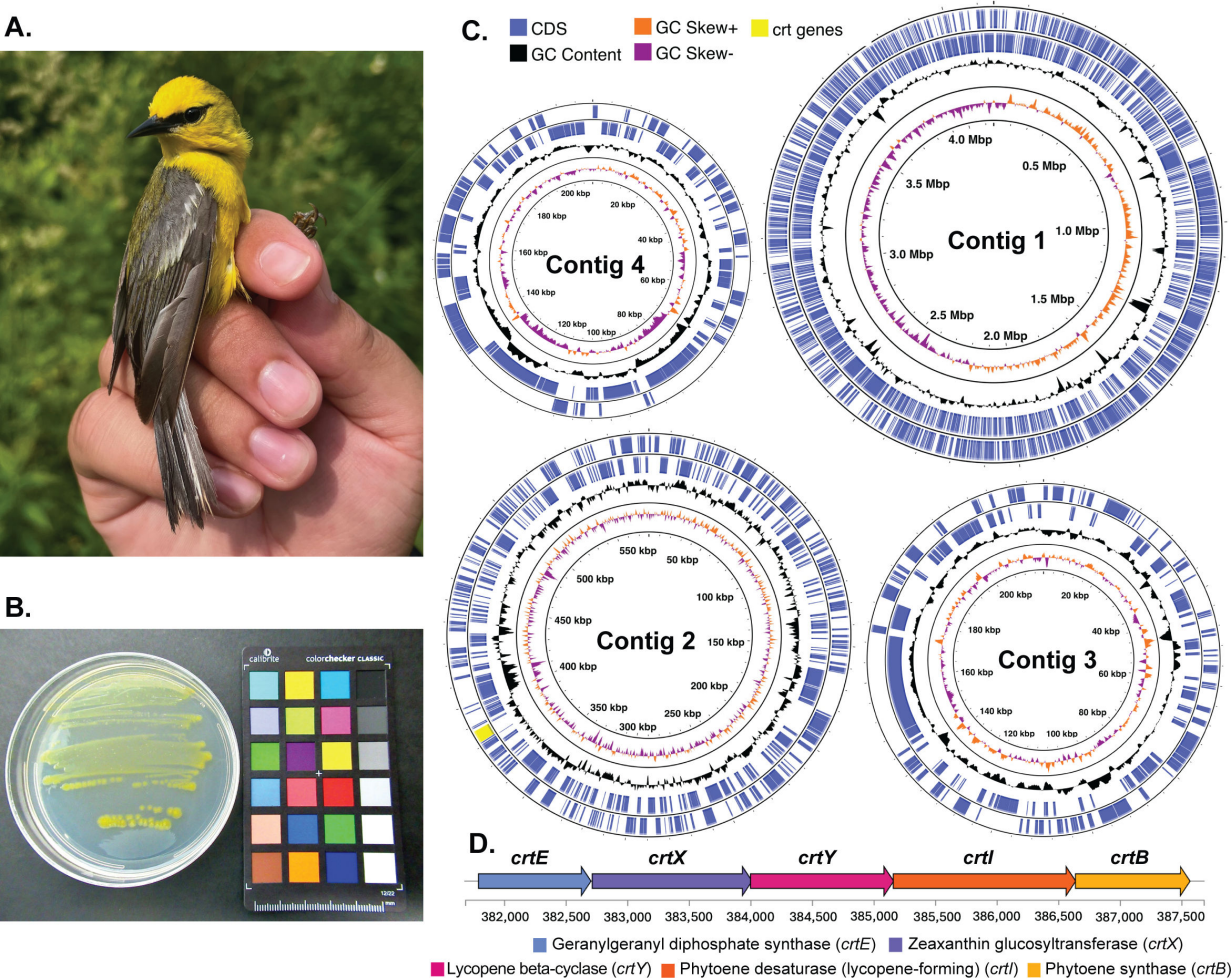
(**A**) Blue-winged warbler (*Vermivora cyanoptera*); (**B**) cultured *Pantoea* isolate and color standard; (**C**) *Pantoea* sp. XAF26B01_ASV70 chromosome (Contig 1) and plasmids (Contigs 2–4); (**D**) *crt* gene cluster on Contig 2

In June 2024, we collected a blue-winged warbler (*Vermivora cyanoptera*) fecal sample in 30% glycerol in phosphate-buffered saline in New York, USA (43.216299,-79.039613; Fig. 1A) under Institutional Animal Care and Use Committee protocol 202400015 by the University at Buffalo. We isolated a yellow-pigmented bacterium on BBL Brain Heart Infusion medium (211059, Beckton Dickinson, Franklin Lakes, NJ, USA), incubated at 35°C for 48 h under aerobic conditions (Fig. 1B). We amplified the full-length 16S rRNA gene (primers 27F/1492R [4]) of the isolate via colony PCR. Amplicons were prepared for sequencing using the Rapid Sequencing V14 Barcoding Kit (SQK-RBK114.96; Oxford Nanopore Technologies [ONT], Oxford, UK) following the manufacturer’s protocol (vRAA_9198_v114_revK_11Dec2024) with minor modifications (half-volume reactions, doubled incubation times). We sequenced 16S libraries on a Flongle Flow Cell (FLO-FLG114; ONT) fitted to a MinION Mk1B device (MIN-101B, ONT). We collected raw data using MinKNOW (v25.03.09), excluding reads with quality scores <9 and lengths <200 bp. We used EPI2ME (v5.2.3) to generate a *de novo* 16S consensus sequence (SRR36621554); default parameters were used except downsampling to 500 reads. A BLASTn search showed that the closest match was *Pantoea agglomerans* strain 1.2.4 (CP134149.1) with 100% sequence identity covering 1476 bp. We used fastANI (v1.2.0) (5) to calculate the average nucleotide identity between CP134149.1 and our isolate, which was 98.4%.

We T-streaked the isolate and extracted genomic DNA (gDNA) from two colonies using the ZymoBIOMICS DNA Miniprep Kit (D4300; Zymo Research, Irvine, CA, USA) following the manufacturer’s protocol. We prepared eight technical replicate gDNA libraries for ONT whole-genome sequencing using the kits, equipment, and modifications described above (vISO_9205_v114_revG_09May2025). We specified super-accurate basecalling (dna_r10.4.1_e8.2_400bps_sup@v5.0.0), trimmed, and demultiplexed raw reads using Dorado (v1.3.0). In Kbase (6), we filtered reads <1 kb using Filtlong (v0.2.1) (7) and performed *de novo* genome assembly and circularization with Flye (v2.9.4) (8). We polished the assembly using Dorado (v1.3.0) and re-oriented contigs to *dnaA* and *repA* using Dnaapler (v1.3.0) (9). We evaluated contamination using CheckM (v1.0.18) (10) and assessed quality with QUAST (v4.4) (11). We identified plasmid sequences using MOB-Suite (v3.1.9) (12) and performed annotation using Bakta (v1.8.2) (13). Default parameters were used except where otherwise noted.

We assessed genome completeness using Benchmarking Universal Single-Copy Orthologs (BUSCO; v5.4.3) (14) with the Enterobacterales data set. The final assembly consisted of a single circular chromosome and three circular plasmids (Fig. 1C). We identified 4,734 coding sequences, including five carotenoid (*crt*) genes clustered on Contig_2 (*crtE, crtX, crtY, crtI, crtB*; Fig. 1D). See Table 1 for genome assembly statistics.

**TABLE 1:** Genome assembly statistics of *Pantoea* sp. XAF26B01_ASV70

Genomic statistics	*Pantoea* sp. XAF26B01_ASV70
Raw sequencing coverage (X) total bases sequenced/total assembly length	39.19
BUSCO completeness (enterobacterales) (%)	99.5
Total BUSCOs used as references from the enterobacterales_odb10 database: 440	99.3% single-copy, 0.2% duplicated, 0.0% fragmented, 0.5% missing
Number of raw reads	65,846
Total bases sequenced	201,947,808
Avg raw read length (bp)	3,067
Total assembly length (bp)	5,153,187
Number of contigs	4
Contig 1/chromosome (bp)	4,147,381
Contig 2/unnamed_1 (bp)	578,806
Contig 3/unnamed_2 (bp)	219,369
Contig 4/unnamed_3 (bp)	207,631
GC content (%)	54.92
Contamination (%)	0.52
N50	4,147,381
*crt* genes	*crtE, crtX, crtY, crtI, crtB*

## Data Availability

This Whole Genome Shotgun project has been deposited at DDBJ/ENA/GenBank under the accession no. JBTNCJ000000000. The version described in this paper is version JBTNCJ010000000.1. The raw sequencing reads are available in the NCBI Sequence Read Archive under the accession SRS27719197, BioSample SAMN54510736, and BioProject PRJNA1399953.
